# Sensitivity and applicability of the Brazilian version of the Brief Assessment of Cognition in Schizophrenia (BACS)

**DOI:** 10.1590/S1980-57642008DN10300007

**Published:** 2007

**Authors:** João Vinícius Salgado, Carolina F. Richards Carvalhaes, Annelise de Matos Pires, Maila de Castro L. das Neves, Breno Fiúza Cruz, Clareci Silva Cardoso, Helio Lauar, Antonio Lúcio Teixeira, Richard S.E. Keefe

**Affiliations:** 1 Department of Psychiatry, Raul Soares Institute, Belo Horizonte, MG, Brazil.; 2 Neuropsychology and Education Department, Health Sciences School, FUMEC University, Belo Horizonte, MG, Brazil.; 3 Epidemiology Research Group.; 4 Neurology Unit, Faculty of Medicine, Federal University of Minas Gerais, Belo Horizonte, MG, Brazil.; 5 Department of Psychiatry and Behavioral Sciences, Duke University Medical Center, Durham, NC, USA.

**Keywords:** schizophrenia, cognition, neuropsychological assessment, BACS

## Abstract

Cognitive assessment in schizophrenia has traditionally used batteries that are long and complex or differ widely in their content. The Brief Assessment of Cognition in Schizophrenia (BACS) has been developed to cover the main cognitive deficits of schizophrenia as well as to be easily and briefly administered, portable, sensitive and reliable.

**Objectives:**

To investigate the applicability and sensitivity of the Brazilian Version of the BACS (Brazilian-BACS).

**Methods:**

Performance of 20 stable patients with schizophrenia on the Brazilian-BACS was compared to 20 matched healthy controls.

**Results:**

Applying the Brazilian-BACS required 43.4±8.4minutes for patients and 40.5±5.7 minutes for controls (p=0.17). All tests demonstrated significant differences between controls and patients (P<0.01). Pearson’s correlation analysis and Cronbach’s a evidenced a high internal consistency for patient performance. The cognitive deficit in the patients was approximately 1.5 standard deviations below controls. These results were consistent with those reported in the validation of the original version and in meta-analyses of similar studies.

**Conclusions:**

The Brazilian-BACS displayed good applicability and sensitivity in assessing the major cognitive constructs that are impaired in schizophrenia. Thus, the Brazilian-BACS seems to be a promising tool for assessing cognition in patients with schizophrenia in Brazil.

## Abstract (pt)

A avaliação cognitiva na esquizofrenia tem utilizado tradicionalmente baterias longas e complexas ou que variam significativamente em seu conteúdo. A “Brief Assessment of Cognition in Schizophrenia” (BACS) foi desenvolvida para cobrir os principais déficits cognitivos na esquizofrenia, para ser de fácil e breve aplicação, assim como portátil, sensível e confiável.

**Objetivos:**

Investigar a aplicabilidade e a sensibilidade da versão brasileira da BACS (Brazilian-BACS).

**Métodos:**

O desempenho de 20 pacientes estáveis com esquizofrenia na Brazilian-BACS foi comparado ao de 20 controles saudáveis pareados.

**Resultados:**

A aplicação da Brazilian-BACS exigiu 43.4±8.4minutos nos pacientes e 40.5±5.7 minutos nos controles (p=0.17). Todos os testes foram significativamente diferentes entre pacientes e controles (P<0.01). A correlação de Pearson e o alfa de Cronbach evidenciaram alto grau de consistência interna no desempenho dos pacientes. O déficit cognitivo nos pacientes foi cerca de 1,5 desvio-padrão menor do que nos controles. Esses resultados são consistentes com os relatados na validação da versão original e em meta-análises de estudos similares.

**Conclusões:**

A Brazilian-BACS mostrou boas aplicabilidade e sensibilidade na investigação dos principais domínios cognitivos comprometidos na esquizofrenia. Portanto, a Brazilian-BACS mostra-se um instrumento promissor no estudo da cognição de pacientes com esquizofrenia no Brasil.

Schizophrenia is characterized by significant and highly disabling cognitive impairment which has a strong impact on functional outcome.^1^ Meta-analyses have shown that schizophrenia is characterized by a broad cognitive impairment with varying degrees of deficit across all domains, measured by standard neuropsychological tests. Impairment is more significant in memory consolidation (spatial and verbal), attention, reasoning and problem solving; while less prominent in visual perceptive abilities, recovery of consolidated memories, procedural memory and reading.^2^

Until recently, however, there was no standard, easily administered brief test battery that specifically and efficiently assesses the cognitive deficits in patients with schizophrenia. Instead, studies assessing cognitive deficits in schizophrenia have used batteries that differ widely in their content of evaluated functions as well as in the specific tests for the same function. Furthermore, many of these are long and complex since they are derived from clinical neuropsychology. These features may represent limiting factors in the evaluation of cognition of schizophrenic patients in clinical practice and therapeutic trials.^3^

These constraints were partly reversed following the development of short batteries assessing cognition in schizophrenia. However, some challenges still remained. Some batteries have their implementation hampered by the requirement of hardware and software,^4,5^ while others lack measures of functions that may be particularly important in schizophrenia, since the batteries were originally developed for other populations.^6-8^

Recently, the Brief Assessment of Cognition in Schizophrenia (BACS) has been developed, with the key features of an ideal battery, including the coverage of main cognitive deficits of schizophrenia, brief administration and scoring time, portability, and reliability. The domains of cognitive function assessed by BACS comprised verbal memory, working memory, motor speed, attention, problem solving and verbal fluency. These domains are consistently impaired and related to outcome in schizophrenia. The battery has two versions (A and B) that differ from each other in terms of alternate forms in those tests that showed a learning effect throughout subsequent administration (verbal memory and Tower of London; see below). The use of alternate versions prevents this learning effect and allows BACS use over time. The BACS is fully portable and designed to be easily administered, requiring 30-40 minutes of testing time with minimal extra time for scoring. The original version of the BACS has been shown to have high test–retest reliability, sensitivity and validity in comparison to a standard battery of cognitive tests.^3^

The availability of a quick and efficient tool like BACS, suitable for use in clinical practice and research settings, may prove extremely useful. BACS may guide clinical decisions on therapeutic interventions to aid cognitive rehabilitation. It can also aid researchers in implementing clinical trials to specifically assess cognitive improvement. These issues are particularly relevant in developing countries due to its low cost, pen and paper format. Thus, this study aimed to evaluate the applicability and sensitivity of the Brazilian version of the BACS (Brazilian-BACS).

## Methods

### Subjects

Twenty patients with a diagnosis of schizophrenia were enrolled in this study. They were recruited from the inpatient and outpatient facilities at Raul Soares Institute, Belo Horizonte, Brazil. The mean time (±SD) of illness diagnosis and treatment was 6.7 (±5.9) years. The mean number (±SD) of psychotic episodes requiring hospitalizations was 3.1 (±3.8). Only one patient was in the first acute episode. The inclusion criteria for patients were: to meet DSM-IV criteria for schizophrenia, to have no history of brain trauma, not to be suffering from a current substance use disorder. The psychiatric diagnosis was performed following a structured clinical interview, MINI-PLUS.^9^ To control the presence of acute psychotic symptoms, only the patients that scored 4 or less in any item on Positive and Negative Symptoms Scale (PANSS), and 19 or less in positive and negative subscales of the PANSS,^10^ were included. Clinically significant extrapyramidal disorders were also excluded by neurological examination and assessed with the Abnormal Involuntary Movement Scale (AIMS).^11^

There were no specific medication criteria for inclusion in the patient group. Six of the 20 patients were being treated with a single atypical antipsychotic medication (three with risperidone, two with olanzapine, one with clozapine), 10 were being treated with a single typical antipsychotic (eight with haloperidol, three with thioridazine), and one with a combination of antipsychotics.

Twenty healthy controls were recruited from a variety of sources. They were required not to have an Axis I disorder according to the DSM-IV criteria based on a structured clinical interview (MINI-PLUS), or any relevant neurological illness.

Patients were interviewed by an experienced psychiatrist that scored symptoms with PANSS and AIMS. Thereafter, patients were randomly assigned to the Brazilian-BACS versions A or B (see below). Control subjects were also interviewed by the psychiatrist and also randomly assigned to the Brazilian-BACS versions A or B.

### The Brazilian Version of the Brief Assessment of Cognition in Schizophrenia (Brazilian-BACS)

The BACS’ adaptation was based on the standard translation/back-translation methodology.^12^ In brief, the original instructions, including the word list, were translated into the Portuguese language by two Brazilian bilingual individuals. The next step was the evaluation of the translation to ensure semantic equivalence and acceptability. Subsequently, the Portuguese version was retranslated into English and compared with the original version.

The constructs measured with the Brazilian-BACS, including the tests, procedures, and measures are listed below in the order administered.

#### Verbal memory

Patients were presented with 15 words and then asked to recall as many as possible. This procedure was repeated five times. There were two alternate forms in versions A and B, respectively. Measure: number of words recalled per trial in any order (range: 0-75).

#### Working memory

Digit sequencing task: Patients were presented with clusters of numbers of increasing length. They were asked to tell the experimenter the numbers in order, from lowest to highest. Measures: number of correct responses (range: 0-28).

#### Motor speed

Token motor task: Patients were given 100 plastic tokens and asked to place them two at a time into a container as quickly as possible. A 60-s time limit was imposed. Measures: the number of tokens correctly placed into the container for the first half-minute, second half-minute, and the 1 min total (range: 0-100).

#### Verbal fluency

Semantic or category fluency: Patients were given 60 s to name as many words as possible within a given category (names of animals).

Phonetic or letter fluency: In two separate trials, patients were given 60 s to generate as many words as possible that begin with a given letter (F, S). Measure: number of words generated per trial.

#### Attention and speed of information processing

Symbol coding: As quickly as possible, patients wrote numerals 1-9 as matches to symbols on a response sheet for 90 s. Measure: number of correct numerals (range: 0-110).

#### Reasoning and problem solving

Tower of London: Patients were shown two pictures simultaneously. Each picture showed three balls of different colors arranged on three pegs with the balls in a distinct arrangement in each picture. Patients were asked to give the total number of times the balls in one picture needed to be moved in order to make the arrangement of balls identical to that of the other opposing picture. There were 20 trials. The items were of variable difficulty, with a general tendency for later items to be more difficult. The test was discontinued if patients made five consecutive incorrect responses. If patients answered correctly for all 20 trials, two additional trials of greater difficulty were administered. There were two alternate forms on versions A and B, respectively. Measure: number of correct responses (range: 0-22).

### Statistical analysis

The variables were processed in a database and statistical analysis carried out using the SPSS 12.0 software. Sensitivity to between-group impairment on all measures was determined with independent t-tests. The internal consistency of the Brazilian-BACS was assessed by Cronbach’s a and the Pearson correlation coefficient calculation. For these analyses, the primary measure from each test of the Brazilian-BACS was standardized by creating z-scores.

## Results

The demographic characteristics of patients with schizophrenia and healthy controls are described in Table 1. The healthy control group was matched to the patient group for education, age and sex.

**Table 1:** Demographics of the sample.

	Schizophrenics			Controls			
	(n=20)			(n=20)			
	Mean	SD		Mean	SD	t	p
Age	32.5	8.8		35.3	12.7	-0.799	0.430
Education (years)	8.4	3.2		9.8	2.4	-1.708	0.096
Sex N (%)	10 (50%)	10 (50%)		10 (50%)	10 (50%)	<0.000	1
PANSS+	14.9	2.4					
PANSS-	15.1	3.1					
AIMS	0.5	0.9					

Table 2 lists the means ±SD for all of the measures on the Brazilian-BACS. All tests demonstrated significant differences between controls and patients (P<0.01). The Brazilian-BACS measures did not differ significantly between males and females in either group. The tests in which there were different forms on versions A and B (i.e. List learning, Tower of London) did not evidence difference between the alternate forms.

**Table 2:** Mean performance of patients with schizophrenia (n=20) and healthy controls (n=20) on the Brazilian version of the Brief Assessment of Cognition in Schizophrenia (BACS).

	Schizophrenics			Controls			
	Mean	SD		Mean	SD	t	p
Verbal memory	27.50	8.62		39.95	6.53	-5,177	<0.001
Digit sequencing	10.55	4.96		17.20	4.35	-4.589	<0.001
Token motor test	50.55	21.02		69.05	12.17	-3.480	<0.001
**Verbal fluency**							
Animals	13.20	3.62		19.35	5.90	-4.079	<0.001
F	9.15	4.22		13.80	3.76	-3.745	=0.001
S	8.40	3.56		12.30	4.24	-3.227	=0.003
Symbol coding	28.75	14.00		48.55	12.25	-4.724	<0.001
Tower of London	7.05	5.12		13.75	4.34	-4.559	<0.001

p, Significance value for patients vs. controls, by t-test.

Table 3 presents the Pearson correlation coefficients for the Brazilian-BACS individual items for schizophrenic and control subjects. The Cronbach’s a value was 0.89.

**Table 3:** Pearson correlation coefficients between individual items of the Brazilian-BACS for patients and controls (n=40).

	Verbal memory	Digit sequencing	Token motor test	Verbal fluency	Symbol coding
Digit sequencing *p*	0.658 <0.001				
Token motor test	0.523	0.541			
*p*	<0.001	<0.001			
Verbal fluency *p*	0.596 <0.001	0.532 <0.001	0.610 <0.001		
Symbol coding *p*	0.556 <0.001	0.627 <0.001	0.636 <0.001	0.641 <0.001	
Tower of London	0.499	0.624	0.556	0.615	0.698
*p*	=0.001	<0.001	<0.001	<0.001	<0.001

The Brazilian-BACS required a mean ±SD 43.4±8.4minutes for patients and a mean ±SD 40.5±5.7minutes for controls (p=0.17).

## Discussion

The Brazilian version of the Brief Assessment of Cognition in Schizophrenia, or Brazilian-BACS, an easily administered pen and paper battery of neurocognitive tests, demonstrated high discriminative power for schizophrenic patients compared to age- and gender-matched controls. This finding cannot be ascribed to the presence of positive, negative or extrapyramidal symptoms since patients with high PANSS scores and significant involuntary movements were not included in this study. Thus, the discriminative property suggests that the Brazilian-BACS is a sensitive tool to evaluate cognitive deficits in schizophrenia and contributes to its construct validation.

All patients were able to complete the tests of the Brazilian-BACS in less than 45 minutes being comparable to the original study. We also found a high degree of internal consistency for the Brazilian-BACS, evidenced by the elevated Cronbach’s a and by the statistically significant correlation between the individual items of the scale on the Pearson correlation analysis.

The magnitude of the cognitive deficit in the patients was approximately 1.5 standard deviations below the healthy controls in this study. These results were consistent with those reported by Keefe et al (2004) in validation of the original BACS version, and in meta-analyses of studies on neurocognitive impairment in schizophrenia.^2^ Nevertheless, it is noteworthy that some biased review studies estimated a more prominent cognitive impairment in schizophrenia.^13^ As argued by Keefe et al. (2004), studies paying special attention to matching groups for age and education may yield differences that are less robust than those that do not incorporate these factors.

Regarding verbal memory and the Tower of London, the versions A and B proved to be very similar in difficulty. As argued by Keefe et al. (2004) the use of alternate forms for these tests is necessary. This prevents learning from the previous administration thereby allowing the assessment of changes over time. The other tests - digit sequencing, symbol coding, verbal fluency, and the token-motor task - have minimal practice effects and so are used without alternate forms. It should also be mentioned that the final measures of the Brazilian-BACS have normal distributed data suggesting minimal ceiling and floor effects. These properties are important for assessing change over time, such as in clinical trials.

It should be noted that our sample was not selected by means of dose medication. Hence, this sample displays the local profile of schizophrenia treatment, characterized by wide range doses of typical or atypical neuroleptics. Typical antipsychotics given at high doses (over 10 mg/day of haloperidol) provide little benefit and may even be deleterious to the cognitive functioning of patients with schizophrenia. Atypical antipsychotic medications seem to improve cognitive performance in schizophrenics, but this effect is evident only when compared to high doses of typical antipsychotics. Lower doses of typical medications may not worsen cognition as do the traditionally used high dose ranges for these medications.^14^ Since in our sample the mean dose (±SD) of typical neuroleptics was 7.7 (±2.4) mg/day and only six patients was using higher doses of typical neuroleptics, it seems unlikely that the cognitive impairment shown in our schizophrenia group was due to antipsychotic medication.

Cognitive impairment in schizophrenia may be present at the first episode of psychosis or at first treatment and its severity remains relatively stable over the lifetime course of illness. This may not be true for the minority of long-term institutionalized poor-outcome patients,^15^ which is not the case of our sample. Thus, the observed cognitive impairment cannot be attributed to a particular feature of the illness stage.

Definitive validation of the Brazilian-BACS requires further study. First, the reliability of Brazilian-BACS measures over time, including its practice effects, must be assessed. As shown by Keefe et al. (2004) the original BACS showed high test-retest reliability over a period of days.

Second, to compare the Brazilian-BACS performance with another instrument known to be sensitive to cognitive impairment in schizophrenia is advisable to perform concurrent validation of the translated battery.

Finally, a complete validation of the Brazilian-BACS requires evaluation of different populations of patients with schizophrenia, including first episode and treatment-refractory patients. It is also important to determine whether Brazilian-BACS scores can predict changes in functional outcome and quality life, and whether the Brazilian-BACS is sensitive to cognitive changes during clinical trials. Studies are underway to determine the validity of the Brazilian-BACS in these areas of inquiry.

In sum, the Brazilian-BACS assesses the major constructs of cognition that have been found to be most impaired and most strongly correlated with outcome in patients with schizophrenia. The Brazilian-BACS takes approximately 40 min to complete in patients with schizophrenia, and yields a high completion rate in these patients. This initial validation makes it a promising tool for assessing cognition repeatedly in patients with schizophrenia in Brazil.

An authorized copy of the test can be obtained by contacting the corresponding author.
